# Arachidonoyl-Specific Diacylglycerol Kinase ε and the Endoplasmic Reticulum

**DOI:** 10.3389/fcell.2016.00132

**Published:** 2016-11-18

**Authors:** Tomoyuki Nakano, Hirooki Matsui, Toshiaki Tanaka, Yasukazu Hozumi, Ken Iseki, Kaneyuki Kawamae, Kaoru Goto

**Affiliations:** ^1^Department of Anatomy and Cell Biology, Yamagata University School of MedicineYamagata, Japan; ^2^Department of Emergency and Critical Care Medicine, Fukushima Medical University School of MedicineFukushima, Japan; ^3^Department of Anesthesiology, Yamagata University School of MedicineYamagata, Japan

**Keywords:** diacylglycerol kinase, arachidonate, substrate specificity, endoplasmic reticulum, phosphoinositide, ER stress

## Abstract

The endoplasmic reticulum (ER) comprises an interconnected membrane network, which is made up of lipid bilayer and associated proteins. This organelle plays a central role in the protein synthesis and sorting. In addition, it represents the synthetic machinery of phospholipids, the major constituents of the biological membrane. In this process, phosphatidic acid (PA) serves as a precursor of all phospholipids, suggesting that PA synthetic activity is closely associated with the ER function. One enzyme responsible for PA synthesis is diacylglycerol kinase (DGK) that phosphorylates diacylglycerol (DG) to PA. DGK is composed of a family of enzymes with distinct features assigned to each isozyme in terms of structure, enzymology, and subcellular localization. Of DGKs, DGKε uniquely exhibits substrate specificity toward arachidonate-containing DG and is shown to reside in the ER. Arachidonic acid, a precursor of bioactive eicosanoids, is usually acylated at the *sn*-2 position of phospholipids, being especially enriched in phosphoinositide. In this review, we focus on arachidonoyl-specific DGKε with respect to the historical context, molecular basis of the substrate specificity and ER-targeting, and functional implications in the ER.

## Introduction

The endoplasmic reticulum (ER), which comprises a tubular and planar network of lipid bilayer membranes (Croze and Morré, 1984), represents a specialized site of protein synthesis and subsequent folding machinery. In addition, the ER plays a central role in the synthesis and transport of major membrane phospholipids such as phosphatidylcholine (PC), phosphatidylserine (PS), and phosphatidylinositol (PI; Gaspar et al., 2007). In response to cellular requirements, this tubular and planar ER network extends to all regions of cell interfaces at membrane contact sites with the plasma membrane, mitochondria, and Golgi apparatus for lipid transfer, integration of metabolic pathways, and calcium homeostasis (Lagace and Ridgway, 2013). In terms of energy homeostasis, fatty acids supply a major source of energy for organisms, but they can also be toxic. When exposed to excess fatty acids, cells esterify fatty acids into neutral lipids and package them into lipid droplets (LDs). Actually, an LD is an ER-derived organelle that is necessary for the storage and mobilization of neutral lipids in a specialized cell type: adipocyte (Martin and Parton, 2006; Brasaemle and Wolins, 2012). Under pathological conditions including nutrient and oxygen starvation, calcium depletion and altered redox status, protein folding, and lipid biosynthesis are impaired, thereby producing ER stress. Therefore, the ER integrates cellular activities of protein and lipid synthesis as well as pathological responses such as unfolding protein response (UPR; Berridge, 2002; Ron and Walter, 2007; Sano and Reed, 2013).

During the process of lipid synthesis, phosphatidic acid (PA) serves as an intermediate molecule for all phospholipids. It is therefore conceivable that PA synthetic activity is intimately involved in the ER function, and that one enzyme responsible for this activity is diacylglycerol kinase (DGK; Kanoh et al., 1990). Actually, DGK comprises a family of enzymes. Each of the isozymes exhibits a characteristic feature in terms of structural, enzymological, and morphological aspects (Goto et al., 2007; Sakane et al., 2007; Mérida et al., 2008; Topham and Epand, 2009; Table 1). Each member of the DGK family presents a unique subcellular localization in transfected cells and presumably plays a specific role at each site (Kobayashi et al., 2007). Of the DGKs, DGKε is unique in its substrate specificity toward arachidonate-containing DG and resides in the ER (Matsui et al., 2014). In this review, we specifically examine the functional role of DGKε in this organelle.

**Table 1:** **Characteristic features of mammalian DGK isozymes**.

	**Molecular weight (kDa)**	**Substrate specificity and Ca^2+^-dependency**	**Main tissue and cell expression**	**Subcellular localization in native cells**	**References**
**TYPE I**					
DGKα	82	Non-specific, Ca^2+^-dependent	T cells, brain (oligodendrocytes)	Cytoplasm, nucleus	Sakane et al., 1990; Schaap et al., 1990; Goto et al., 1992
DGKβ	90	Non-specific, Ca^2+^-dependent	Brain (striatal neurons)	Perisynaptic membrane	Goto and Kondo, 1993; Hozumi et al., 2008
DGKγ	88	Non-specific, Ca^2+^-dependent	Brain (cerebellar Purkinje neurons)	Golgi apparatus	Goto et al., 1994; Kai et al., 1994; Nakano et al., 2012
**TYPE II**					
DGKδ	130	Non-specific	Reproductive organs, leukocytes, ubiquitous	Cytoplasm	Sakane et al., 1996, 2002
DGKη	127	Non-specific	Reproductive organs, ubiquitous	Cytoplasm	Klauck et al., 1996; Murakami et al., 2003
DGKκ	142	Non-specific	Reproductive organs	Plasma membrane	Imai et al., 2005
**TYPE III**					
DGKε	64	sn-2-arachidonoyl (20:4) -DG-specific	Brain (neurons), ubiquitous	Endoplasmic reticulum	Lemaitre et al., 1990; Tang et al., 1996; Shulga et al., 2011a; Matsui et al., 2014
**TYPE IV**					
DGKζ	104	Non-specific	Brain (neurons), ubiquitous	Nucleus	Bunting et al., 1996; Goto and Kondo, 1996; Hozumi et al., 2003
DGKι	117	Non-specific	Brain (neurons), retina	Postsynaptic region of rod bipolar dendrites	Ding et al., 1998; Ito et al., 2004; Hozumi et al., 2013
**TYPE V**					
DGKθ	110	Non-specific	Brain (neurons), smooth muscle, and endothelial cells	Excitatory presynapses, nuclear speckles	Houssa et al., 1997; Walker et al., 2001; Tabellini et al., 2003; Goldschmidt et al., 2016

## Identification of arachidonoyl DGK

Since the first discovery of DGK activity in a brain microsome fraction (Hokin and Hokin, 1959), it has been reported as distributed widely in animal tissues (Hokin and Hokin, 1963; Sastry and Hokin, 1966; Prottey and Hawthorne, 1967; Lapetina and Hawthorne, 1971; Farese et al., 1981). The DGK activity was associated with various fractions of cells, including soluble, membranous, and cytoskeletal fractions (Call and Rubert, 1973; Daleo et al., 1974). These features suggest the heterogeneity of DGK in animal tissues and cells. In this respect, Glomset group reported that cytosolic and membrane-bound DGKs in Swiss 3T3 cells show different substrate selectivity (MacDonald et al., 1988). Intriguingly, the membrane-bound DGK is unique in that it selectively catalyzes DG containing arachidonate at the *sn*-2 position (Lemaitre et al., 1990). Moreover, it is rapidly inactivated by preincubation with its preferred substrate. Generally, DGK activity is determined using several assay systems with different detergents, including octylglucoside mixed micelle assay, deoxycholate assay, and Triton X-100 assay (Walsh et al., 1994). It is particularly noteworthy that the detection of arachidonoyl-specificity depends on the assay system that is used. The substrate selectivity toward arachidonate-containing DG is most sensitive in the octylglucoside assay, but is not detected in the deoxycholate assay. The sensitive assay system together with presumed thermal lability made it difficult to purify the enzyme. Biochemical purification of this “arachidonoyl DGK” from bovine testis estimated the molecular mass as 58,000 (Walsh et al., 1994), although PCR cloning using degenerate primers succeeded in isolating the cDNA clone encoding arachidonoyl DGK, designated as DGKε (Tang et al., 1996).

## Molecular basis for arachidonoyl specificity of DGKε

DGKε is the only isozyme that shows substrate specificity toward arachidonate (20:4)-containing DG. As a substrate for DGKε, *sn*-1-stearoyl-2-arachidonoyl-DG (18:0/20:4-DG) is preferred over saturated DG (*sn*-1,2-didecanoyl-DG, 10:0/10:0-DG) or monounsaturared DG (*sn*-1,2-dioleoyl-DG, 18:1/18:1-DG). It should be mentioned that DGKε prefers 18:0/20:4-DG to *sn*-1-stearoyl-2-linoleoyl-DG (18:0/18:2-DG) and *sn*-1-stearoyl-2-docosahexaenoyl-DG (18:0/22:6-DG) (Lemaitre et al., 1990; Tang et al., 1996; Shulga et al., 2011a). Therefore, it is concluded that DGKε prefers arachidonate at the *sn*-2 position. Arachidonic acid, an essential polyunsaturated fatty acid, contains four double bonds. Arachidonate is not only a major component of membrane phospholipid; it is also the precursor of bioactive molecules designated as eicosanoids, such as prostaglandins and leukotrienes that are catalyzed, respectively, by cyclooxygenase (COX) and lipoxygenase (LOX; Funk, 2001; Buczynski et al., 2009). Because these arachidonate-derivatives serve as key mediators of several pathophysiological events, free arachidonate itself should be maintained within a restricted concentration (Peters-Golden and Henderson, 2007). Under physiological conditions, arachidonate is incorporated into the *sn*-2 position of phospholipids by the enzymes arachidonoyl-CoA synthetase and lysophospholipid acyltransferases (Pérez-Chacón et al., 2009). These enzymes, together with DGKε, specifically recognize arachidonate moiety. However, how is the arachidonoyl specificity achieved?

To investigate the molecular basis of the substrate specificity of DGKε toward arachidonate, the Epand group compared amino acid sequences of the enzymes that specifically recognize this fatty acid (Shulga et al., 2011b). They identified in the catalytic domain of DGKε (aa. 421–453 in human sequence) the motif L-X(3-4)-R-X(2)-L-X(4)-G, in which -X(n)- is n residues of any amino acid. This domain, which is contained in DGKε of various species as well as phosphatidylinositol-4-phosphate-5-kinase type Iα, resembles a polyunsaturated fatty acid-recognizing domain identified in lipoxygenases (Neau et al., 2009). Mutations of the essential residues in this motif, L431I and L438I, significantly reduce arachidonoyl specificity. Furthermore, the group found a sequence similar to this LOX-like motif in non-specific isozyme DGKα, with a V656 residue instead of Leu in DGKε. They confirm that V656L mutation introduces some specificity for arachidonate-containing DG to DGKα.

## Targeting of DGKε to the ER

The DGK family is localized to distinct subcellular compartments in cDNA-transfected cells, including the cytoplasm, ER, Golgi complex, actin-cytoskeleton, and nucleus (Kobayashi et al., 2007). In an early fractionation study using Swiss 3T3 cells, “arachidonoyl DGK activity” comigrated with that of the ER marker enzymes, together with other PI-metabolizing enzyme PI synthase (Glomset, 1996). In agreement with the biochemical data, DGKε is targeted to the ER. Because DGKε is highly insoluble, the hydrophobic region was presumed to play a key role in the ER targeting. We investigated the sequence responsible for ER targeting of DGKε (Matsui et al., 2014). Various deletion and substitution mutations of rat DGKε tagged with GFP were transfected in cells and were compared with ER markers. Results show clearly that a stretch of hydrophobic amino acid sequence 20–40 (DGKε 20–40) in the N-terminus is a determinant sequence in controlling the ER targeting of DGKε. This hydrophobic region adopts an α-helical structure of the transmembrane segment (Glukhov et al., 2007).

In this regard, a detailed modeling study suggests the possibility that this sequence structure can take two representative models of low-energy conformations, such as a long straight helix and a U-bend helix (Decaffmeyer et al., 2008). Two interchangeable structures of monotopic and bitopic nature might confer on DGKε a unique feature in relation to the ER. Changing conditions such as a redox state and pH can regulate the conformation of DGKε between these two structures, thereby affecting the relation of DGKε and the ER membrane.

**Figure 1:**
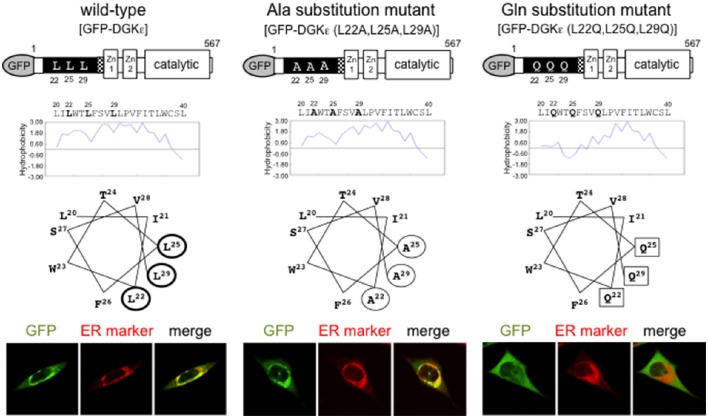
**Features of wild-type DGKε and Ala and Gln substitution mutants**. Hydropathy plot analysis and helical wheel projections of the hydrophobic region of DGKε are shown. Wild-type DGKε contains highly hydrophobic residues L22, L25, L29 (thick circles), which represent a prominent hydrophobic patch. Alanine substitution mutant DGKε (L22A, L25A, L29A) have a reduced hydrophobic patch (thin circles), whereas glutamine substitution mutant DGKε (L22Q, L25Q, L29Q) includes a hydrophilic patch (squares). Immunofluorescence images of GFP for full-length wild-type and substitution mutants of DGKε together with ER marker staining in transfected HeLa cells (lower panels). Wild-type DGKε and alanine mutant DGKε (L22A, L25A, L29A) clearly colocalize with ER marker, whereas glutamine mutant DGKε (L22Q, L25Q, L29Q) shows diffuse cytoplasmic pattern. Modified from Matsui et al. (2014) with permission from Elsevier.

The α-helical structure of the hydrophobic sequence 20–40 creates a “hydrophobic patch” composed of L22, L25, and L29 (according to the rat sequence; Figure 1). To test whether the hydrophobicity is critical in the ER targeting, we produced two substitution mutants: one containing less hydrophobic Ala (hydrophobic score 1.8) and the other with hydrophilic Gln (hydrophobic score -3.5), instead of wild-type Leu (hydrophobic score 3.8; (Matsui et al., 2014)). Ala substitution fragment DGKε (20–40/L22A, L25A, L29A) is targeted to the ER membrane. It is recovered in the membrane fraction, along with wild-type fragment. However, Gln substitution fragment DGKε (20–40/L22G, L25G, L29G) containing a “hydrophilic patch” is distributed diffusely in the cytoplasm and is recovered in the soluble fraction. Furthermore, full-length Ala mutant DGKε (L22A, L25A, L29A) is shown to reside in the ER whereas Gln mutant DGKε (L22G, L25G, L29G) abolishes it. These findings suggest that the hydrophobic patch composed of L22, L25, and L29 is crucially important for ER targeting of DGKε.

No general consensus sequence for ER localization, such as the ER retention signal, is found in this hydrophobic or in other regions of DGKε (Matsui et al., 2014). Therefore, details of the ER targeting mechanism of DGKε remain unclear. How does this hydrophobic patch specifically lead DGKε to the ER, instead of the other membranes such as mitochondrial membrane? The ER consists of phospholipid bilayer containing a plethora of proteins. Is the membrane or the protein of the ER recognized by the hydrophobic patch? Does the hydrophobic patch bind to some microdomain of the membrane? Because DGKε-kinase dead mutant also resides in the ER, the substrate DG and the product PA are not involved in subcellular localization of DGKε. Therefore, the current data can be summarized as follows: ER targeting is mediated through the N-terminal hydrophobic patch composed of L22, L25, and L29. Subsequent recognition of the arachidonoyl acyl chain of DG is achieved by a LOX-like motif in the catalytic domain of DGKε (aa. 421–453). Additional studies must be conducted to elucidate the ER targeting mechanism of DGKε.

## ER stress

ER homeostasis is crucially important for cellular activity and survival (Ellgaard and Helenius, 2003). Stress in the ER induces the UPR, which represents a complex signaling system that controls translation and transcription in response to increased demands on the protein folding capacity of the ER for cell survival (Rutkowski and Kaufman, 2004; Koumenis and Wouters, 2006; Malhotra and Kaufman, 2007; Hetz, 2012). To meet this demand, the UPR coordinates membrane growth and phospholipid metabolism, thereby leading to ER membrane expansion and enhanced protein folding capacity (Sriburi et al., 2004). In addition to misfolding or incomplete assembly of proteins, alteration of the ER lipid composition also is shown to initiate ER stress (Devries-Seimon et al., 2005), indicating that disruption of membrane lipid homeostasis triggers directly or indirectly a mechanism to reestablish ER lipid composition (Fagone and Jackowski, 2009).

Under ER stress conditions, the glucose-regulated protein GRP78 plays a key role in UPR (Bertolotti et al., 2000). GRP78, a member of the heat shock protein 70 superfamily, serves as a major ER chaperone protein with ATPase activity. It is a key regulator of the transmembrane ER stress sensors comprised of inositol requiring enzyme 1 (IRE1), protein kinase RNA-activated (PKR)-like ER kinase (PERK), and activating transcription factor-6 (ATF6) (Lee, 2014). IRE1 is a transmembrane ribonuclease that splices and activates X-box-binding protein (XBP-1) mRNA. Spliced form of XBP-1 [XBP-1(S)], together with cleaved ATF6 and ATF4, translocates to the nucleus where they induce the expression of ER stress-responsive genes (Hetz, 2012). In this regard, XBP-1(S) serves as a regulator of PC synthesis and ER membrane development (Fagone and Jackowski, 2009). PERK mediates activation of the pro-apoptotic factor C/EBP homologous protein (CHOP), thereby leading to apoptosis if the response is insufficient to reestablish homeostasis (Xu et al., 2005; Shore et al., 2011).

Therefore, the ER membrane expansion is supported by phospholipid synthesis, in which PA serves as an intermediate product. The initial step in the PA synthesis is catalyzed using a family of glycerol 3-phosphate acyltransferases located in the ER and the outer mitochondrial membrane, followed by acyl-CoA-dependent acylation of lyso-PA to form PA (Lagace and Ridgway, 2013).

Another intermediate product DG is a precursor for PA, which is catalyzed by DGK. Therefore, DGK is intimately involved in phospholipid synthesis in the ER and presumably in the UPR. Earlier, we examined whether ER-resident DGKε participates in this process and assessed the ER stress pathways in DGKε knockdown cells under experimental ER stress conditions using tunicamycin and thapsigargin (Matsui et al., 2014). From DGKε deletion experiments conducted under ER stress conditions, we found the following: (1) The major protein chaperone GRP78 is induced to the same extent in both wild-type and DGKε-deficient cells. (2) Eukaryotic initiation factor 2α (eIF2α) is slightly, although not significantly, downregulated at the total and phosphorylated protein levels. (3) CHOP is significantly suppressed at the protein level. Analysis of cellular vulnerability, however, clearly shows that DGKε deletion reduces cell viability under ER stress conditions to some degree. Therefore, DGKε deletion seems to exert conflicting effects on apoptosis in terms of CHOP expression. In this regard, recent studies suggest that although CHOP is identified originally as a repressive member of the C/EBP family of transcription factors (Ron and Habener, 1992), it is capable of either transcriptional repression or activation, depending on the context (Oyadomari and Mori, 2004). Further studies need to be done to elucidate this point.

## Concluding remarks

Gene duplication contributes to the evolution of living creatures by expanding DNA information. *Escherichia coli* is equipped with two forms of DGK (Van Horn and Sanders, 2012; Jennings et al., 2015) whereas mammalian cells contain at least 10 isozymes. In the course of evolution, one branch of the diversified DGKs might have gained substrate specificity toward arachidonate-containing DG. Of DGs, arachidonoyl DG is phosphorylated selectively by arachidonoyl DGK to produce corresponding PA, which is further incorporated into inositol phospholipids. Multiple steps of this process are expected to enrich arachidonate in PI (Glomset, 1996). Because PIP2 is a major substrate for PLC, its enzymatic action results in the production of arachidonoyl DG. Functional implication of arachidonoyl DGK is suggested by an experimental seizure model at the organismal level. It reveals that DGKε-KO mice show lower degradation of brain PIP2 and lower accumulation of arachidonoyl-DG and free arachidonate although resting levels of PIP and PIP2 are similar between wild-type and DGKε-KO mice brains (Rodriguez de Turco et al., 2001). These findings suggest that DGKε is clearly involved in recycling PI metabolism presumably through the enrichment of arachidonate moiety. Therefore, DGKε downregulation may lead to a slowdown of PI turnover, thereby downregulating various membrane functions. In addition, it is particularly noteworthy that arachidonoyl DG also represents a substrate for another enzyme DG lipase, which cleaves *sn*-1-acyl chain to produce 2-arachidonoyl glycerol (Maejima et al., 2005). Because 2-arachidonoyl glycerol serves as an endocannabinoid for retrograde synaptic transmission, arachidonoyl DG is located at the crossroad of the two signaling cascades: DG-PA and DG-2AG pathways directed respectively by DGK and DG lipase. The mechanisms for how these signaling pathways are coordinated in parallel are just beginning to be explored.

## Author contributions

TN, HM, TT, YH, KI, and KK did the experiments in the original papers and summarized the results for the mini review. TN and KG constituted and wrote the manuscript.

### Conflict of interest statement

The authors declare that the research was conducted in the absence of any commercial or financial relationships that could be construed as a potential conflict of interest.
